# Sustainable and smart rail transit based on advanced self-powered sensing technology

**DOI:** 10.1016/j.isci.2024.111306

**Published:** 2024-11-05

**Authors:** Hongjie Tang, Lingji Kong, Zheng Fang, Zutao Zhang, Jianhong Zhou, Hongyu Chen, Jiantong Sun, Xiaolong Zou

**Affiliations:** 1 School of Information Science and Technology, Southwest Jiaotong University, Chengdu 610031, P.R. China; 2 Chengdu Technological University, Chengdu 611730, P.R. China; 3 School of Mechanical Engineering, Southwest Jiaotong University, Chengdu 610031, P.R. China; 4 School of Design, Southwest Jiaotong University, Chengdu 610031, P.R. China

**Keywords:** Artificial intelligence, Engineering, Mechanical engineering, Energy systems

## Abstract

As rail transit continues to develop, expanding railway networks increase the demand for sustainable energy supply and intelligent infrastructure management. In recent years, advanced rail self-powered technology has rapidly progressed toward artificial intelligence and the internet of things (AIoT). This review primarily discusses the self-powered and self-sensing systems in rail transit, analyzing their current characteristics and innovative potentials in different scenarios. Based on this analysis, we further explore an IoT framework supported by sustainable self-powered sensing systems including device nodes, network communication, and platform deployment. Additionally, technologies about cloud computing and edge computing deployed in railway IoT enable more effective utilization. The deployed intelligent algorithms such as machine learning (ML) and deep learning (DL) can provide comprehensive monitoring, management, and maintenance in railway environments. Furthermore, this study explores research in other cross-disciplinary fields to investigate the potential of emerging technologies and analyze the trends for future development in rail transit.

## Graphical abstract

**Figure:**
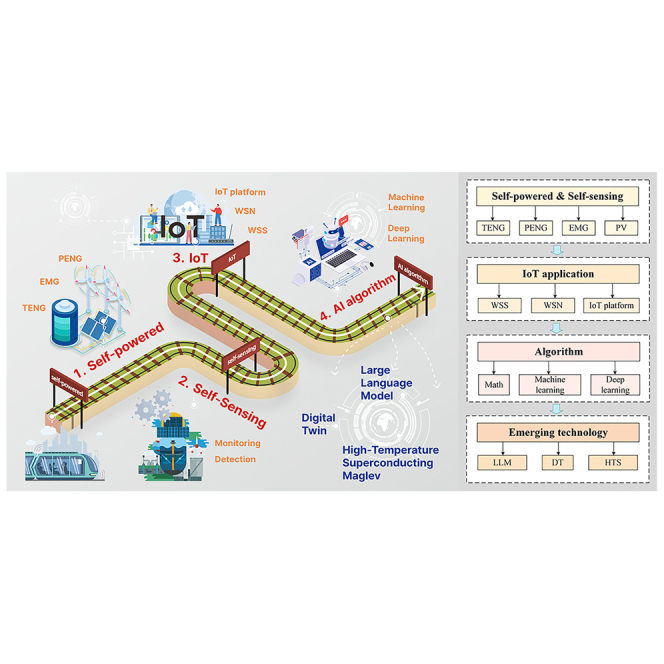

## Introduction

Rail transit, which includes high-speed railways, subways, light rail, and other urban rail networks, plays an essential role in daily transportation activities. The enclosed tracks of rail transport ensure safety, economic efficiency, and reliability, thereby significantly promoting inter-city economic development and reducing traffic congestion. The global railway network is expanding rapidly, with an average of 6,600 km of new lines added annually, including 3,000 km of high-speed rail in China alone.^1^ However, the increasing number of railway kilometers, higher passenger speeds, and more considerable cargo tonnage, have led to a growing demand for continuous maintenance in rail transit. Nevertheless, the development of self-powered technology, the IoT, artificial intelligence (AI), and other emerging technologies offers more energy-efficient, intelligent ways to maintain railway infrastructure.

The rapid development of railways has led to the widespread application of large-scale signal and sensor networks in rail transport systems. However, their substantial energy requirements significantly increase overall operational costs. Monitoring sensors rely on batteries due to the lack of onboard power sources and spatial constraints. The extensive maintenance and replacement of these batteries escalate costs and environmental issues, making them unsuitable for intelligent transportation’s low-cost and sustainable energy needs.^2^ Therefore, harnessing clean and renewable energy from the rail environment for application in railway vehicles and tracks holds immense potential.^3^^,^^4^ During railway operations, numerous studies have shown that moving vehicles can directly provide various high-energy density, highly available, and environmentally friendly mechanical energy sources.^5^^,^^6^ Collecting energy from renewable sources to supplement or replace batteries is currently the most feasible strategy for achieving long-term management of railway wireless detection devices.

The development of intelligent railway transportation requires a reliable energy supply and increases the demand for monitoring and trackside electronic equipment.^7^ Real-time, continuous, and effective condition monitoring of railway vehicles and track environments through various sensors (optical, ultrasonic, infrared, and acoustic sensors) are key features of the gradual intelligence enhancement of railways.^8^ The current focus of railway IoT technology development primarily lies in a comprehensive IoT architecture, which includes wireless smart sensors (WSS), wireless sensor networks (WSN), and remote cloud monitoring platforms.^9^^,^^10^ Self-powered railway IoT application have significant potential for development. For example, it no longer relies solely on single sensors but integrates installed energy harvesting devices for vehicle or environmental data collection and communication.^11^ It achieves intelligent sensing nodes.^12^ Furthermore, by examining the evolution of sensing and communication technologies within the railway IoT,^13^ sensor network development in this context will enter a new phase that encompasses wider detection ranges and more innovative management methods such as data analysis, low-power wide-area networks (LPWAN),^14^ and artificial intelligence (AI) technologies.

With the continuous, stable, and sustainable energy supply from self-powered devices,^15^ intelligent algorithms deployed for optimizing and monitoring rail transit can adapt to changing environments and improve performance. Many algorithms directly process the electrical signals generated by the power supply devices, performing tasks such as recognition, classification, and anomaly detection. This enables secondary energy utilization, known as self-powered sensing. Machine learning (ML) and deep learning (DL) algorithms, which are subsets of AI, play a predominant role in this process.^16^ The advancements in information and communication technologies (ICT), such as 5G communication networks, have enhanced data collection rates to meet the requirements for extensive data analysis and more sophisticated AI applications.

Since the initial exploratory stages a few years ago,^17^ these algorithms, surpassing traditional methods’ capabilities, have undergone evolved significantly to conduct complex and comprehensive analyses of system-acquired datasets. ML algorithms have already demonstrated successful applications in traffic flow prediction, traffic congestion modeling, and scheduling tasks such as mode selection.^18^^,^^19^^,^^20^^,^^21^ In rail transit, emerging evidence indicates these algorithms’ vast potential.^22^ Their applications include analyzing vehicle equipment conditions, detecting driver states, monitoring surrounding environment safety, optimizing traffic control, and managing energy. Additionally, emerging technologies in other fields^23^ such as maglev technology, large language models (LLM), and digital twin (DT) also provide broader perspectives for the rapid development direction of large-capacity and intelligent rail transit.

**Figure 1:**
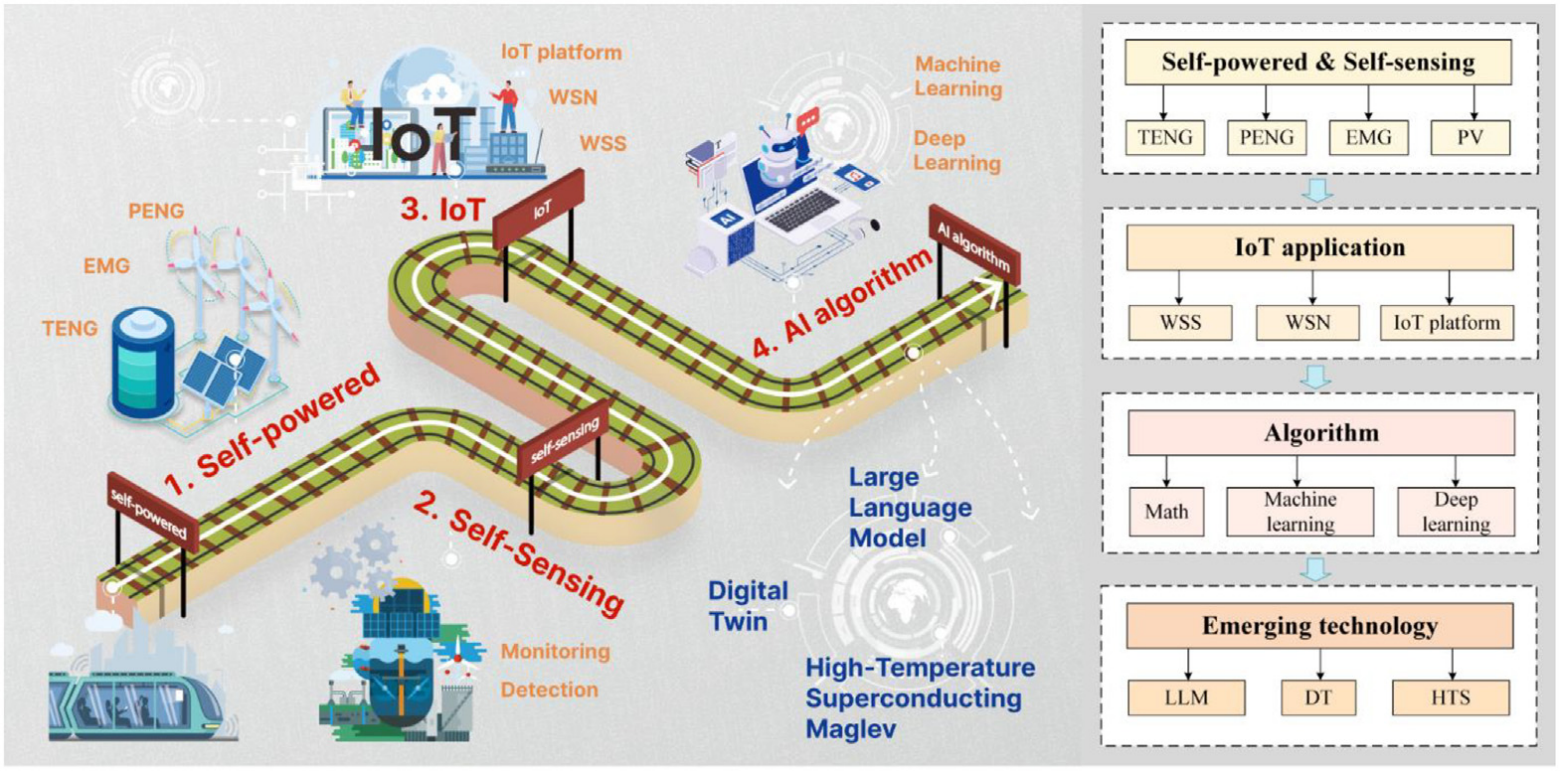
Sketch and flowchart of AIoT applications and emerging technologies in sustainable and smart rail transit

According to Industry 4.0 technologies, the integration of AI and IoT, known as AIoT, has been extensively applied in rail transit^24^ and has become crucial for the realization of future intelligent autonomous train operations (iATO).^25^ As shown in Figure 1, this review mainly discusses the traditional self-powered, self-sensing devices used in various rail scenarios, analyzing their characteristics, innovations, and development directions. It further conducts a hierarchical analysis of advanced technologies like the IoT, introducing the role of self-powered sensing devices in the railway IoT framework. Additionally, this review analyzes various algorithms, including modeling, ML, and DL that widely used in different rail scenarios. It also highlights their features, advantages, and innovative applications. Finally, from the perspective of emerging AIoT technologies, this review explores interdisciplinary research in this field, analyzing potential trends for future intelligent development of rail transit.

## Self-powered sensing technology

Self-powered devices have significantly developed, resulting in various designs and implementations widely applied in rail transit. These devices collect energy from different scenarios within this field.^26^ As shown in Table 1, the collection methods include triboelectric nanogenerators (TENGs),^27^^,^^28^ which convert mechanical vibrations into electrical energy through contact electrification and separation; piezoelectric nanogenerators (PENGs),^29^^,^^30^ which utilize the property of piezoelectric materials to generate charges under mechanical stress, it is suitable for harvesting vibrational energy; and electromagnetic generators (EMGs),^31^^,^^32^^,^^33^ which rely on electromagnetic induction, generating current through the relative motion between coils and magnets; Wind turbines can also generate power by harnessing the airflow created by moving trains. Other natural energy sources can be harvested through photovoltaic cells,^34^ pyroelectric nanogenerators,^35^ and acoustic energy collectors.^36^

**Table 1:** Comparison of several energy harvesting technologies

	Triboelectric nanogenerator (TENG)	Electromagnetic generator (EMG)	Piezoelectric (PZT)	Photovoltaic (PV)
Principles	Triboelectric effect and electrostatic induction.	Faraday’s laws of electrolysis.	The asymmetric movement of charges in piezoelectric material when subjected to mechanical strain.	Electron-hole pair generated from the semiconductor materials.
Efficiency	high area power density and energy conversion efficiency.	High conversion efficiency, high current, low resistive impedance.	High voltage and power density.	Depends on the materials.
scalability and applicability	Affected by humidity and other environmental factors. Apply to most scenarios, such as irregular and low-frequency mechanical energy collection.	Apply to most scenarios.	Affected by the range of working frequency. Normally being integrated to MEMS.	Affected by solar radiation and temperature. Apply to transportation equipment, household & building systems, and others.

These self-powered systems demonstrate significant potential in enhancing energy efficiency, reducing maintenance costs, and promoting green transportation in rail systems. They can be deployed in various scenarios of rail transit, sustainably powering remote monitoring devices, signaling systems, and sensor networks. It also serves as an auxiliary energy source for train operation and maintenance, thus supporting the development of intelligent transportation systems. Numerous studies have highlighted innovations in power generation modes, scenarios, and materials of these self-powered devices, marking them as critical drivers for sustainable development in rail transit.

**Figure 2:**
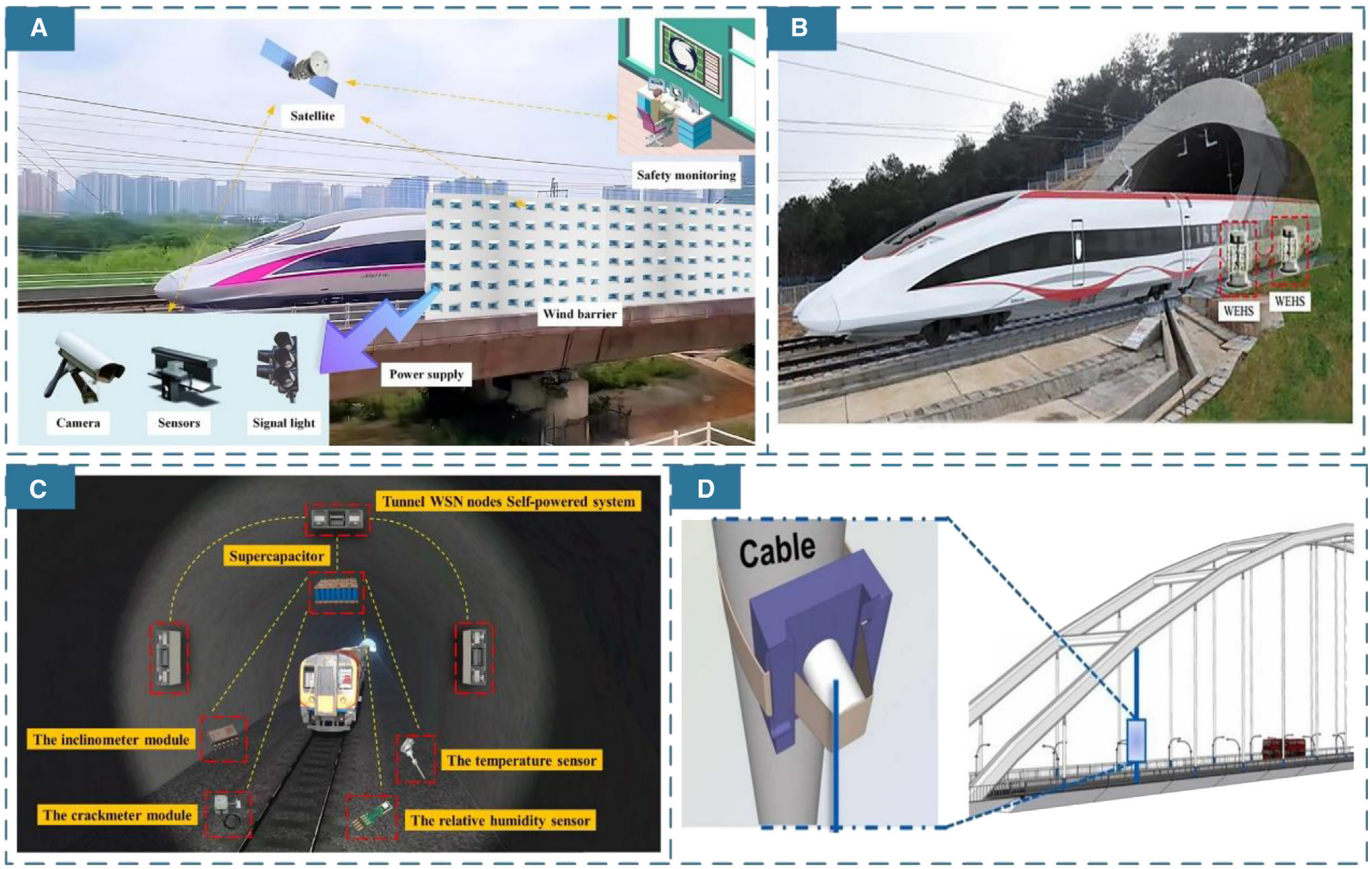
Railway energy harvesters in different scenarios (A) Wind barrier with sensors beside the railway. (Elsevier Publishing.^40^ Reproduced with permission. All rights reserved.). (B) Wind energy harvesters at the mouth of the tunnel. (Elsevier Publishing.^42^ Reproduced with permission. All rights reserved.). (C) Various sensors and self-powered systems are installed in the tunnel. (Elsevier Publishing.^43^ Reproduced with permission. All rights reserved.). (D) TENG sensors on the bridge cables for structural inspection. (Elsevier Publishing.^45^ Reproduced with permission. All rights reserved.).

Currently, typical applications of energy harvesters in rail transit include collecting mechanical energy from train components such as suspensions, bogies, wheel bearings, and surrounding tracks.^37^ Research has developed additional features for these scenarios, like energy-saving damping, condition monitoring, and road improvement.^38^ Compared to regular railways, high-speed rail systems require more stringent maintenance and foreign object detection on the catenary system. For instance, Zheng et al. focused on the catenary system of high-speed railways, detecting foreign object intrusion to reduce energy loss and prevent damage to overhead contact lines.^39^ Figure 2A shows Xiong et al.’s proposal to install wind barriers along the tracks, designed to address the risks that windy conditions pose to vehicles operating on high-speed rail and the challenges faced by sensors in maintaining sustainable operations. This innovative wind barrier structure not only mitigates the effects of crosswinds on trains but also harnesses wind energy to power sensors, thereby establishing a sustainable system for detecting wind speed safety within the rail environment.^40^^,^^41^ Furthermore, there are various self-powered sensor devices for monitoring and maintaining other infrastructure along train routes, such as tunnels and bridges. Figure 2B demonstrates Pan et al.’s utilization of the piston effect generated by high-speed trains passing through tunnels to collect renewable wind energy and power sensors in the remote rail scenarios.^42^ The proposed S-rotor and H-rotor collectors can efficiently capture wind energy, thereby facilitating the sustainable monitoring and maintenance of sensors in the tunnel. This approach ultimately contributes to the long-term safety of high-speed train operations. Figure 2C presents Zheng et al.’s novel tunnel WSN node self-powered system, which facilitates the real-time monitoring of the safety characteristics of tunnel structures. The system employs electromagnetic induction and piezoelectric patches to harvest wind energy. This hybrid energy collection methods enables the device to effectively capture wind energy under both high and low wind speed conditions, thereby addressing the power supply challenges faced by safety monitoring systems in subway tunnels.^43^ For bridge structures, self-powered devices enable real-time monitoring and assessment, identifying potential issues for timely maintenance and repairs.^44^ Figure 2D introduces Huang et al.’s self-driving acceleration TENG sensor on bridge cables, which is employed for real-time monitoring of infrastructures, including railway tracks on bridges. The sensor captures vibration signals and structural acceleration responses, integrating TENG technologies into structural health monitoring to facilitate continuous long-term monitoring of the bridge cable structure in real time.^45^

**Figure 3:**
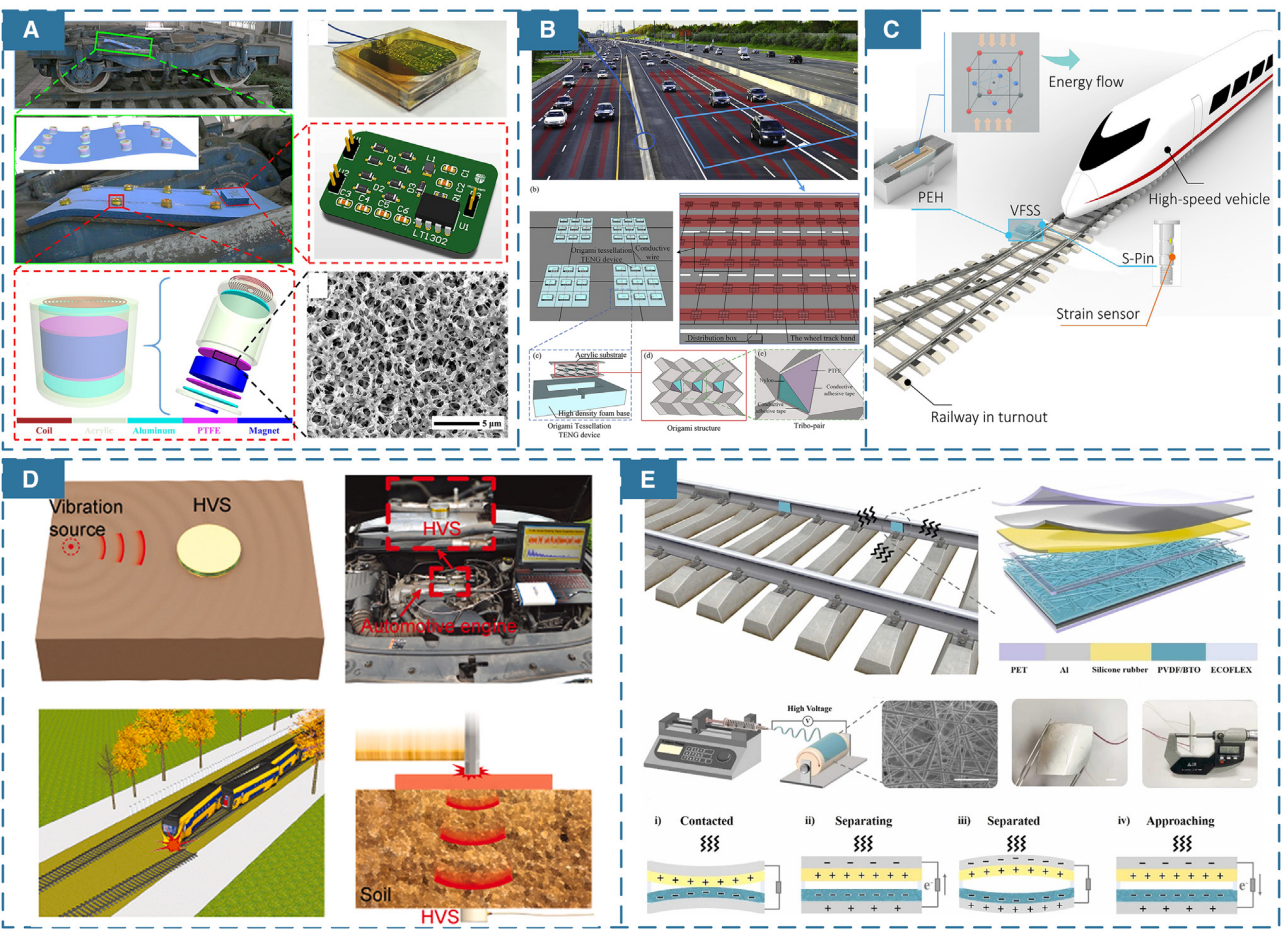
Diversification of materials for self-powered devices (A) A maglev porous nanogenerator with porous structure and bendability. (Elsevier Publishing.^47^ Reproduced with permission. All rights reserved.). (B) Origami tessellation (OT) based TENGs with multi shapes and multi layers facets. (Elsevier Publishing.^48^ Reproduced with permission. All rights reserved.). (C) A block-shaped piezoelectric transducer that stacks multiple chemical film units. (Elsevier Publishing.^49^ Reproduced with permission. All rights reserved.). (D) Self-powered wideband vibration sensor with layer-powder-layer structure. (Elsevier Publishing.^50^ Reproduced with permission. All rights reserved.). (E) Self-powered vibration sensors for TENG based on electrospinning nanofibers. (Elsevier Publishing.^51^ Reproduced with permission. All rights reserved.).

The diversification of application scenarios has spurred extensive research into improving materials for self-powered devices. In rail transit, developing advanced multifunctional sensing and self-powered flexible devices is an approach to practically strengthen the versatility of self-powered systems and thus overcome corresponding limitations.^46^ For instance, as shown in Figure 3A, Jin et al.^47^ proposed a magnetic levitation porous nanogenerator (MPNG) by improving material applications, making the self-powered device flexible and better suited for train bogies. The porous structure of the new materials provides a rough surface, enhancing the stability and sustainability of energy harvesting. Therefore, MPNG arrays supply sustainably power that enables the transmission of real-time data to other devices, achieving the railway monitoring. Moreover, inspired by the increased surface area of origami multilayer structures, Zhang et al.^48^ developed a Paper TENG (P-TENG) by altering the materials, which provided an effective topology for triboelectric energy harvesting, as illustrated in Figure 3B. P-TENG can significantly improve the output performance of TENGs across a broader range of geometric configurations and in more adaptable environments for vibration energy harvesting.

Beyond adapting devices to application scenarios, many studies have focused on improving the energy-harvesting efficiency of these devices.^52^^,^^53^^,^^54^ For example, in Figure 3C Sun^49^ proposed a battery-free vibration-powered force sensing system (VFSS) which integrates structural loading, sensing, and energy harvesting. The system also utilized chemical synthesis of new materials which could sense tensile and compressive forces, to integrate strain film sensors into bearings. The proposed VFSS is able to provide real-time data for the safe operation and continuous monitoring of railway switch machine systems. Thus, material innovations also endow self-powered devices with enhanced functionality. Lin et al.,^50^ as shown in Figure 3D, designed a self-powered high-frequency vibration sensor (HVS) with a layer-powder-layer structure. HVS enhances the response to a wider frequency range of track vibrations, providing diverse applications such as burst vibration detection, rail track fracture identification, automobile engine monitoring, and more. In Figure 3E, Yan et al.^51^ developed an electrospinning nanofiber PENG sensor on rail fasteners. The system not only harnesses rail vibration energy to achieve sustainable power supply, but also accurately assesses the tightness of rail fasteners based on their vibrational characteristics. This dual functionality provides more detection methods for maintenance and is essential for ensuring the safe operation of railway.

Despite numerous innovative studies, most current research treats self-powered devices only as power sources. The lack of versatility in self-powered devices results them unsuitable for integration, which leads to low utilization including a certain waste of space and cost when devices are not working. However, energy harvesting is a multidisciplinary technology involving materials, mechanics, electronics, and control. Cross-disciplinary research can expand the functionality of self-powered devices.^55^^,^^56^ In particular, self-sensing applications, which utilize the voltage and current signals directly generated by self-powered devices, have been widely discussed in rail transit and other self-powered scenarios. Self-sensing applications provide intuitive and accurate condition detection of trains and their surroundings. With the support of emerging ICT, including the IoT framework and AI algorithms, the collected voltage and current data from self-sensing can be applied to create more intelligent and efficient self-powered nodes. For sustainable rail development, self-powered sensing technology must be foundational to advance the field and enable intelligent applications.

## Advanced IOT applications

IoT technology has developed various rail-specific applications with extensive functionalities. These applications encompass logistics, management, maintenance, monitoring, and control systems for railways,^8^^,^^46^ aiming to enhance the sustainability of railway management and operations.^10^ Sensors enabled by the development of the IoT can be deployed in any location and obtain more information and data in high quality from the sensors network. However, the expanding capacity of IoT introduces several challenges: insufficient functionality of individual sensor nodes, poor coordination between these sensors, inadequate energy supply, high operation and maintenance costs, and issues with the quality of service in wireless transmission networks.^57^ Unstable and inefficient IoT components can pose significant risks and impacts on rail transit, especially concerning railway, electrical, and maintenance safety.

**Figure 4:**
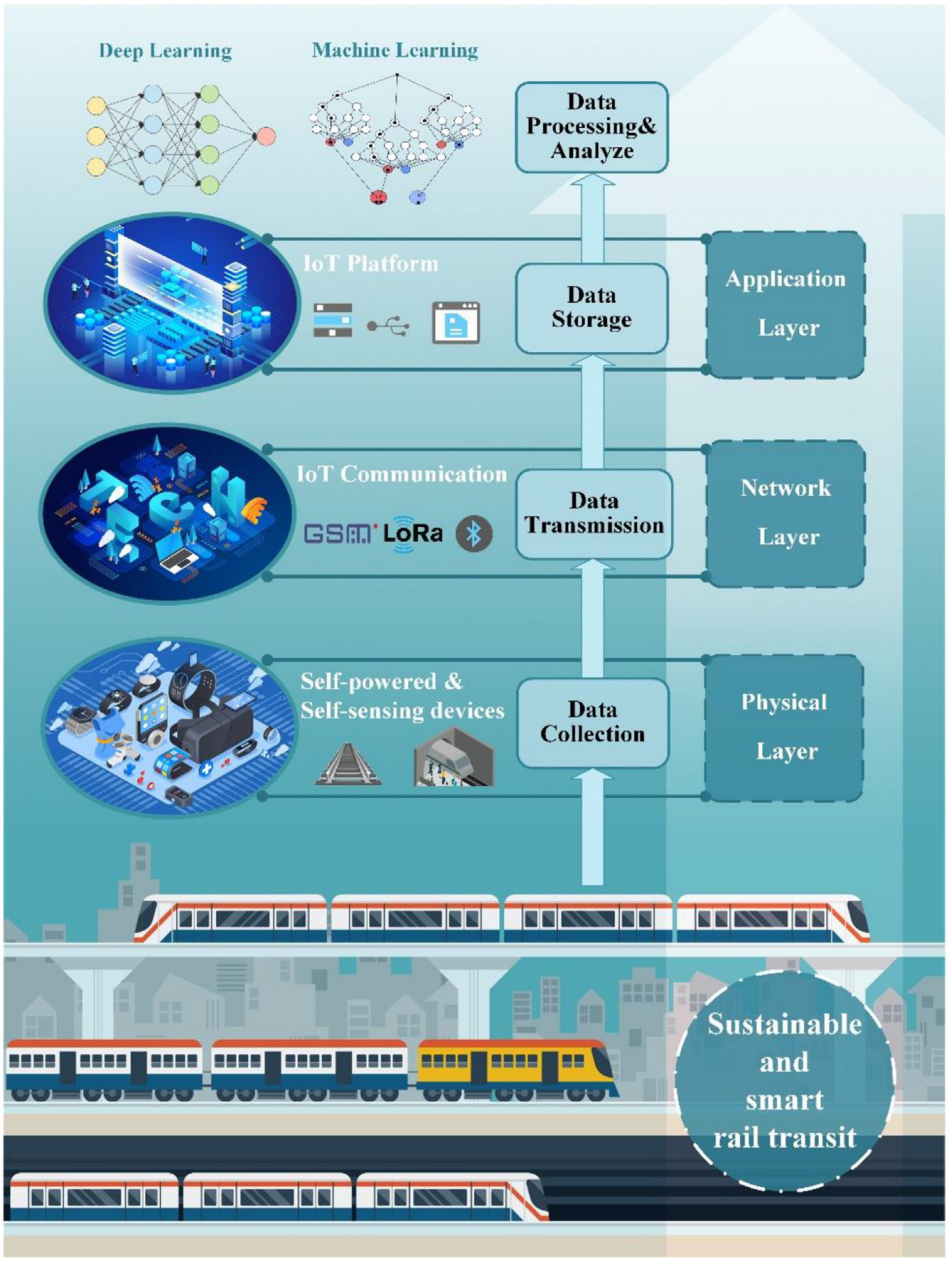
A comprehensive IoT structure applied in sustainable and smart rail transit

Thus, integrating previously discussed self-powered and self-sensing technologies is essential for the sustainable and green development of railway IoT.^58^^,^^59^^,^^60^ Various energy harvesting and sensing technologies enable sensors or IoT applications to achieve self-sufficiency or self-support.^11^ Liu et al.^61^ discussed several self-sustaining IoT systems, covering energy harvesters, sensing devices, and signal transmission systems. These devices and systems leverage technologies within extensive railway infrastructure to reduce their life cycle costs. Simultaneously, railway facilities can employ IoT applications for preventive maintenance, enhancing rail transit safety and efficiency. Yang et al.^12^ described a more comprehensive IoT architecture, including the structure from the physical layer, network layer, to the application layer. This chapter will also reference such architectures, organizing the applications of various self-powered and self-sensing devices within IoT. As shown in Figure 4, the analysis will follow the composition of IoT, focusing on wireless smart sensors (WSS), wireless sensor networks (WSN), and cloud or local terminal data storage.

### Wireless smart sensors (WSS)

Previous studies^62^^,^^63^^,^^64^ have extensively discussed the composition of sensor networks in railway IoT. Wireless sensor nodes based on various sensors are the constituent units of WSN, and the basic components of the IoT. Typically, wireless sensor nodes are deployed on infrastructure or base stations and integrate multiple types of sensors, mobile computing processors, and communication modules. With the advancement of micro-electro-mechanical systems (MEMS),^65^ sensor nodes within the IoT architecture for rail transit can be fully integrated with energy harvesters. These nodes can also become intelligent through self-powered and self-sensing modes, enhancing their functionality. This section will discuss self-powered WSS in rail transit, comparing and analyzing the features of sustainable, intelligent sensor nodes regarding application scenarios and functionality.

**Figure 5:**
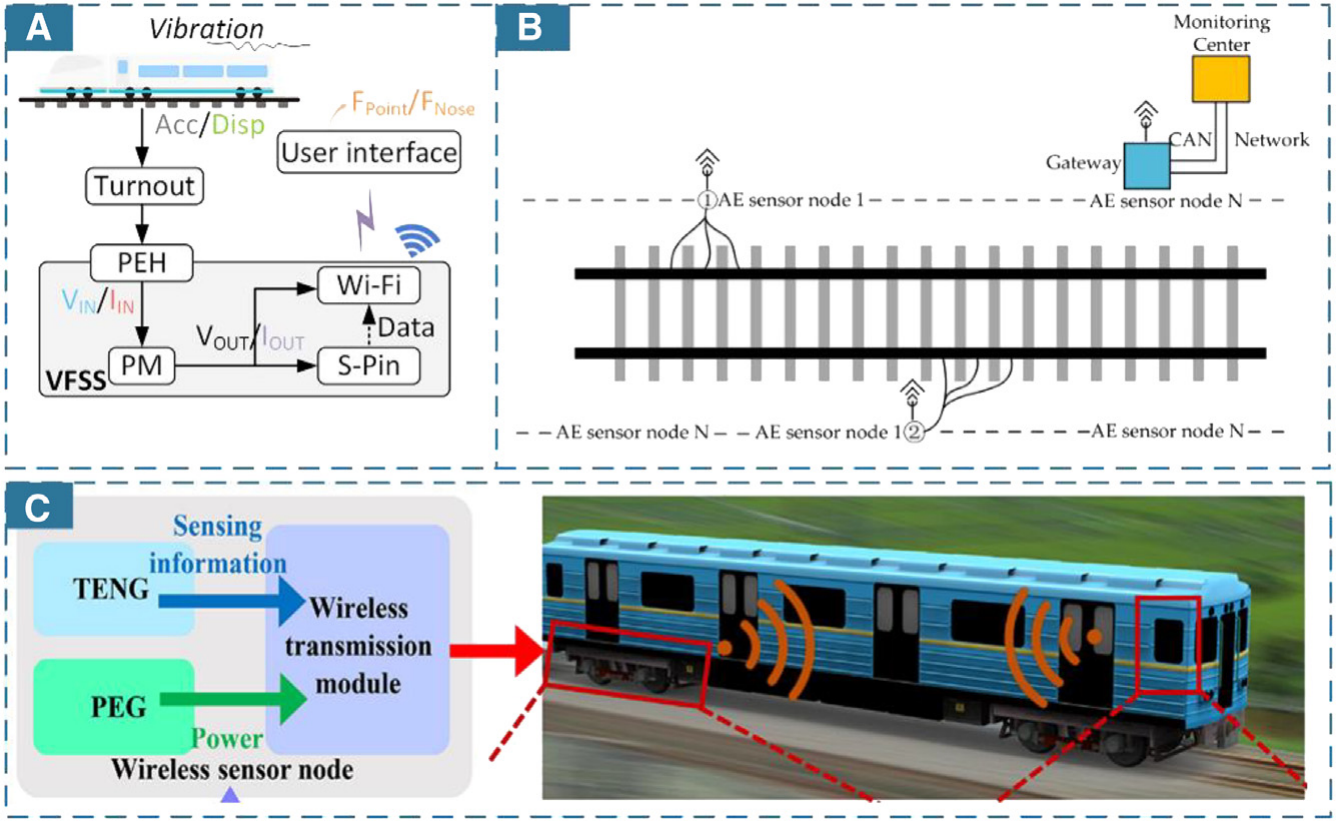
WSS applications in different railway scenarios (A) Integrated intelligent components with stress, signal and energy flow functions. (Elsevier Publishing.^49^ Reproduced with permission. All rights reserved.). (B) WSS monitors structural vibration response. (Elsevier Publishing.^71^ Reproduced with permission. All rights reserved.). (C) Low-power WSS design is used for abnormal vibration monitoring of various equipment. (Elsevier Publishing.^72^ Reproduced with permission. All rights reserved.).

Adequate energy supply allows individual devices to be independently and appropriately embedded in various application scenarios. Flexible applications mean more data can be collected and monitored while rail vehicles operating. Some studies have installed wireless sensor nodes on train wheel axles.^66^^,^^67^ For example, Gong et al.^68^ embedded a variable resistor energy harvester (VREH) into train wheel bearings as WSN nodes to continuously monitor bearing conditions. Figure 5A shows nodes embedded in tracks and railway turnouts, using stress sensors to monitor structures while collecting vibration energy for wireless data transmission.^49^^,^^69^ This setup forms an integrated WSS that collects stress, signal, and energy flows. Thus, the proposed system developed an autonomous, battery-free, and sustainable monitoring node. Wang et al.^70^ embedded a WSN node into a train coupling scheme, integrating a processor, energy harvester, sensor, and signal transmitter to create an in-train forces monitoring system. As wireless sensors become more intelligent, IoT nodes must provide more functionalities beyond simple monitoring. Most self-powered WSSs unfortunately rely on data collected by individual sensors. However, integrating energy harvesters, sensors, and wireless transmission modules into a single self-powered WSS can yield more accurate data for various state detections in rail transit. Figure 5B Wu^71^ proposed a wireless acoustic sensor network (WASN) for rail flaw detection and localization diagnosis system. The WASN nodes rapidly sample the acoustic emission signals on the rail surface, subsequently calculating and wirelessly transmitting their characteristic parameters to the gateway for data processing. Wang et al.^72^ designed a self-sustained WSS node based on a hybridized TENG and piezoelectric energy generator (PEG) vibration mechanism. It includes a microcontroller unit (MCU) and a radio frequency (RF) transceiver for continuous monitoring of track vibration signals in harsh environments, as illustrated in Figure 5C. In the proposed self-sustainable node, the output from the TENG serves as the acceleration sensing signal, which is subsequently processed by the MCU. Meanwhile, the PEG provides a continuous power supply to both MCU and RF transceiver, enabling effective transmission of the acceleration signal. Cii et al.^73^ shows a node powered by solar panels, designed with integrated power management, data acquisition, and wireless transmission circuits. This node transmits its generated voltage signals to the onboard control unit, synchronizing the monitoring and controlling the train’s status.

### Wireless sensor network (WSN)

WSNs comprise spatially distributed independent sensor nodes.^62^ The dynamic nature of the environment in the rail transit necessitates the construction of more stable and secure WSNs^74^ to enable unified, efficient, and intelligent monitoring and management of embedded devices. In addition to WSS’s self-sufficiency and intelligent management capabilities, communication technology advancements have expanded WSN coverage and adaptability to various operational environments along railway lines. This section will discuss the improvements, innovations, and development trends in WSNs in Industry 4.0 that provide fundamental communication functionalities within rail transit by integrating self-powered and self-sensing devices.

**Figure 6:**
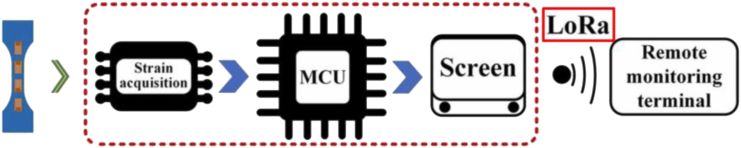
WSN applications with self-powered devices in railway: Processed voltage data is transmitted through the LoRa network (Elsevier Publishing.^82^ Reproduced with permission. All rights reserved.).

Li designed a^75^ battery-free railway track defect monitoring system, which utilizes radio frequency energy to collect and transmit railway health status data to a remote-control center via the global system for mobile communications railway (GSM-R) communication system. Bi^76^ proposed a TENG-driven self-powered sensor node that utilizes long time evolution (LTE) network communication for long-distance wireless transmission of abnormal sound signals. In addition to wide-area mobile communication networks such as GSM-R, LTE-R, and 5G-R,^77^^,^^78^^,^^79^ LPWANs like long range low power radio frequency technology (LoRa) and narrowband internet of things (NB-IoT) are also commonly used for communication between rail transit sensor nodes and remote terminal servers.^80^ Quevy et al. used a TENG energy harvester to power acoustic sensors.^81^ Considering the power limitations of energy harvesting, the focus was on the power consumption of protocols and node communication within the sensor network. LoRa networks and cloud applications were chosen for communication within embedded gateways, transmitting data about traffic signals. Gao et al.^82^ constructed an integrated sensor node that processes collected voltage data in the MCU, identifying loads and calculating damage from strain signals. Data are then transmitted via a LoRa wireless module for remote terminal monitoring of structural fatigue, as depicted in Figure 6.

For the control of trains and local monitoring using sensor data, constructing a highly mobile vehicle ad-hoc network (VANET) for local area network communication^83^^,^^84^ is essential to ensure data privacy and security. These networks are well-suited for long-term monitoring in challenging environments, as they can overcome transmission delays and data loss.^85^ Bluetooth is often used as a convenient and relatively low-power short-range local communication method for point-to-point transmission of data collected by self-powered sensor nodes in rail environments.^66^ Zanelli demonstrates solar panels mounted outside train carriages to power sensors and communication modules. Bluetooth low energy (BLE) boards receive data from wireless sensor nodes and are stored locally.^13^ Other short-range communication networks such as Zigbee, WIFI, and radiofrequency identification (RFID) also have widespread applications.^86^^,^^87^^,^^88^ For the performance and characteristics of different communication methods, Table 2 shows several specific comparative results.

**Table 2:** Comparison of different IoT communication networks and protocols based on self-powered systems

References	Self-powered harvesters	Communication	Distance	Power consumption	Application/Target
Wang et al.^86^	EMG	Zigbee	10-100 m	Very low consumption	Transmit data from sensors mounted on the wheels to a monitoring terminal
Zanelli et al.^13^	Photovoltaic generator	Bluetooth Low Energy 5.0(BLE) /4G/LTE	10-100 m	Very low consumption	Build a gateway, communicate with wireless sensor nodes through BLE in LAN; Data is transmitted to a remote PC via a 4G/LTE modem within the WAN
Dziadak et al.^66^	PENG	BLE/WIFI	10-100 m	Very low/Higher consumption	The transmission protocol is selected according to the power generation, so that different modes of measurement data communication can be carried out
He et al.^89^	EMG	Wireless Transceiver	10-100 m	Low consumption	The vibration profile parameters collected by the remote data logger are transmitted for train positioning and axle safety monitoring
Quevy et al.^81^	TENG	LoRa/NB-IoT/EnOcean/Miwi	1-10 km	Very low consumption	A new traffic signal control method is designed by communicating with the remote server while tracking the vehicle
Gong et al.^68^	Variable reluctance energy harvester (VREH)	Wireless Transceiver（RF）	1-100 m	Adjustable consumption	The recorded temperature, vibration and strain are stored on an SD card and then transmitted to a PC via RF signals for monitoring
Magno et al.^88^	Photovoltaic generator	Bluetooth/Zigbee	2-30 m	Low consumption	Design wireless video sensor nodes with local processing, low-power hardware, power management, and energy harvesting
Li et al.^75^	Radio-frequency energy harvester	GSM-R	1-10 km	Relatively high	The power supply and track health status are collected through RF and the railway status data is transmitted to the control center
Bi et al.^76^	PENG	LTE	1-10 km	Lower than GSM, higher than short-haul communication	The track data is transmitted wirelessly to the remote server through LTE for foreign object detection
Cii et al.^73^	Photovoltaic generator	Zigbee	10-100 m	Very low consumption	All measured values are collected and the microprocessor properly analyzes the data.
Sun et al.^49^	PENG	WIFI	100-300 m	Higher consumption	The track vibration signal collected by the energy collector is transmitted wirelessly to the client through WIFI
Balid et al.^59^	EH Unit	Zigbee	10-100 m	Very low consumption	Use the advantages of a mesh network topology, thereby eliminating a single point of failure.

### IoT platform

From a technical perspective, the IoT platform is situated at the application layer of the architecture, also known as the application enablement platform (AEP). It encompasses an operable upper-layer infrastructure and provides service endpoints for all data collected by sensor nodes. With the increasing support for WSS and WSN from self-powered and self-sensing devices, a growing volume of sensor data demands enhanced device management, data security, and information regulation on the IoT platform. Automated and continuous remote monitoring significantly reduces labor and time costs. Currently, some railway self-powered IoT applications transmit data via WSN to local servers or edge devices for storage or edge computing. For example, He et al.^90^ designed a self-powered railway wagon monitoring sensor which is powered by EMG technology and mounted on the carriages of freight trains and is utilized to collect monitoring data from freight cars. The data are subsequently read and analyzed by the embedded algorithm in edge device MCU, thereby facilitating the detection of axle cracks. However, considering the computational limitations and performance constraints of embedded devices, many studies transmit data over wide-area networks to remote terminals for processing, computation, and monitoring,^91^^,^^92^ thus leveraging cloud computing applications.

**Figure 7:**
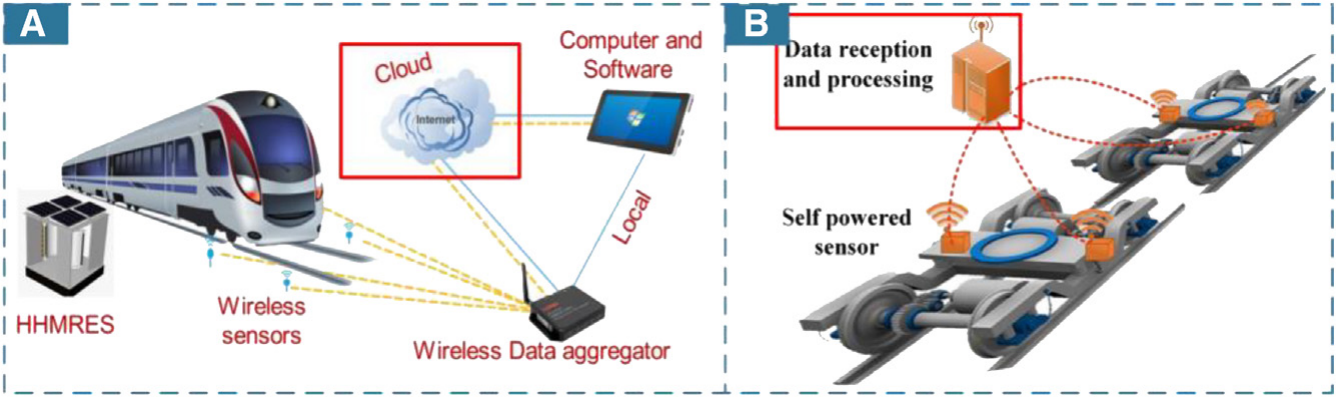
Cloud or local IoT platform based on self-powered devices (A) Cloud processing and local monitoring of hybrid multimodal renewable energy harvesting system (HHMRES). (Elsevier Publishing.^93^ Reproduced with permission. All rights reserved.). (B) Local data reception and processing of data from self-powered sensors. (Elsevier Publishing.^91^ Reproduced with permission. All rights reserved.).

In terms of the scalability of IoT platform functionalities, both on-premises and cloud-based servers can achieve intelligent operations by incorporating data analysis or algorithmic capabilities beyond simple data monitoring. Cloud-based deployment facilitates easier application expansion through algorithmic software, and online maintenance also reduces costs,^12^^,^^93^^,^^94^ as depicted in Figure 7A. Gao et al. transmitted raw data to the cloud, where cloud-deployed algorithms identify foreign object intrusion and detect faults in rail transit scenarios.^95^ Local deployments retain real-time solid performance and high security. Related research focuses on embedding ML algorithms in local gateways or servers to enable faster anomaly detection across various scenarios,^13^^,^^67^^,^^91^ as shown in Figure 7B. Chen focused on providing various functions on edge devices, using local inference on data to detect anomalies.^96^ He argues that local deployment minimizes maintenance work and data transmission costs while end-to-end inference reduces potential data security issues.

From an architectural perspective, the IoT encompasses the physical, network, and application layers, which correspond to the discussed WSS, WSN, and IoT platform components. The use of self-powered and self-sensing devices has led to various advancements in rail transit IoT technology. Self-powered technology provides renewable energy for sensor devices in railway scenarios while self-sensing technology offers diverse types of sensor data in the rail environment. The combination of these technologies enables greener and more sustainable vehicle and environmental monitoring. However, further functional expansion in applications is necessary to meet the intelligent demands of Industry 4.0. The IoT platform has already provided an essential environment for deploying intelligent algorithms. Therefore, researching the application and development of ML and AI algorithms is crucial for achieving intelligent AIoT in rail transit IoT.

## Advanced algorithms

Self-powered and self-sensing technologies offer promising energy solutions for rail transit. The IoT application in railways has led to more stable and diverse sensor data collection in the track environment. As a result, advanced self-powered systems demonstrate significant potential in both the front-end and back-end intelligent applications of the system.^97^

The evolution of algorithms has always accompanied the development of intelligent transportation and smart railways. Traditionally, data analysis and statistics were primarily handled through mathematical modeling.^98^ However, with continuous algorithmic improvements and enhanced computational capabilities, an increasing number of self-powered scenarios now support the deployment of AI algorithms, including machine learning (ML) and deep learning (DL) applications.^99^ ML enables computational devices to mimic human behavior, thereby automating and intelligently processing data to some extent.^100^^,^^101^ Popular ML algorithms include Support Vector Machines (SVM), Decision Trees (DT), and Random Forests. SVM is a supervised learning algorithm, that excels at solving nonlinear classification problems in small to medium-sized datasets such as anomaly detection.

In recent years, the rapid development of DL, particularly with artificial neural networks (ANN), has brought it closer to achieving AI. Supervised learning in DL significantly enhances model training.^22^ For instance, convolutional neural networks (CNNs), primarily used for feature extraction in computer vision (CV), exhibit strong generalization capabilities for classification tasks across various scenarios. CNNs, with relatively simple model structures and parameters, can handle images or complex multi-channel data collected by sensors in harsh environments. Additionally, recurrent neural networks (RNNs) like long short-term memory (LSTM) and gated recurrent unit (GRU) networks excel at processing long-sequence signals, such as long-term voltage power sequence signals generated by TENG sensors deployed in the field. These RNNs capture sequential relationships and positional information in time series, addressing long-distance dependencies and accurately identifying device status changes over time. Furthermore, advanced Transformer networks, known for their success in natural language processing (NLP), also utilize self-attention mechanisms for regression prediction in general time series, enabling routine anomaly state warnings.

Currently, the application of algorithms in rail transit research has developed a relatively mature system.^16^ On one hand, ML is grounded in fundamental mathematical principles, which facilitates the deployment of algorithms in a wide range of application scenarios. On the other hand, DL with its modular network architecture, can achieve more sophisticated and intelligent functionalities. Consequently, it is reasonable for the widespread application of advanced algorithms in railway transportation, including track environment monitoring, train status recognition, vehicle optimization management, and driver behavior analysis. However, research and improvement of algorithms are still in their infancy, whether for the control of rail vehicles^102^ or the maintenance and fault detection of infrastructure.^103^ Present applications often overlook the importance of cross-disciplinary approaches. Due to the compatibility constraints within a single algorithm, many studies struggle to effectively adapt algorithms across diverse scenarios. Therefore, it is essential to consider the practical deployment of the algorithm on embedded devices, in order to more effectively overcome the challenges in real-world implementation. For instance, Chen et al.^96^ proposed a low-cost system based on the self-powered sensors and tiny ML techniques. Several algorithms and trained models like RF and deep neural network (DNN) are deployed on the embedded system. The low-cost MCU can run on-device ML models at a milliwatt-level power consumption, solving problems of high power-consumption and insufficient computing memory. He also employed datasets collected under normal and abnormal track conditions to train a network, ultimately detecting various abnormal states in the track environment.

This chapter analyzes algorithm examples from various research scenarios, providing new evidence that emerging intelligent algorithms, when combined with self-powered, self-sensing technologies and IoT applications, offer significant advantages and promising prospects.

### Anomaly detection for rail environment

The maintenance costs of rail transit infrastructure, which include components such as tracks, sleepers, signal controllers, and transformers, constitute a significant portion of total expenses, typically ranging from 20% to 30%.^104^^,^^105^ The application of algorithms is particularly important in reducing the human and material resources required for track maintenance and management. By deploying intelligent algorithms in self-powered systems, continuous operation can be achieved, enabling effective monitoring and preventive maintenance of infrastructure across various scenarios.^106^

Common monitoring and maintenance measures include detecting defects or cracks in the tracks and monitoring for foreign object intrusion or anomalies on the tracks. Table 3 presents examples of self-powered, self-sensing methods and algorithm types used in different environmental anomaly detection scenarios.

**Table 3:** Algorithms and their features on self-powered devices

References	Self-powered	Self-sensing	Algorithm	Features of Algorithm	Application
Lu et al.^69^	EMG	__	Littlewood-Paley wavelet analysis	For processing non-stationary signals and can provide both time and frequency information	health monitoring of urban rail corrugation
Meng et al.^51^	TENG	Voltages	Features analysis of original voltage signals	Extract key features of the voltage signal, such as amplitude, frequency, and phase	Rail fasteners tightness safety detection
Alavi et al.^107^	PENG	Voltages	Three-dimensional finite element model analysis	Complex three-dimensional physics problems are simulated numerically	Road Detection
Bi et al.^76^	TENG	__	Analysis of sensor capacitance changes resulting from by the sound signals	Measure the effect of sound signal on sensor capacitance	Railway foreign matter intrusion detection
Magno et al.^88^	Photovoltaic generator	__	CV image processing: Background subtraction	Remove the static background from the video frame to highlight moving objects	Abandoned/Removed object detection
Chellaswamy et al.^108^	__	__	Particle Swarm Optimization(PSO)	An optimization algorithm based on swarm intelligence	Railway track irregularity detection
Sun et al.^109^	PENG	Voltages	Multi-stable phase trajectories and wavelet analysis	The behavior trajectories of the system in different stable states are analyzed for nonlinear dynamic system analysis	Rail corrugation detection
Wang et al.^7^	EMG	Voltages& Electromotive forces	Short Time Fourier Transform (STFT) analysis	Fourier transform is applied to the local time period of the signal	Rail corrugation detection
Sun et al.^110^	EMG	Voltages& Electromotive forces	Time-frequency wavelet analysis	The combination of time and frequency analysis provides time-varying frequency characteristics of the signal	Rail corrugation detection
Wang et al.^111^	EMG	__	Stack Denoising Autoencoder (SDA)	Multiple Denoising Auto encoders are stacked together to form a deep architecture	Transformer fault detection
Kamakshi et al.^84^	PENG	__	Collision Avoidance Algorithm	Distance measurement and threshold crossing	Train collision prevention

Given that vibration signals from mechanical equipment are typically nonlinear and non-stationary, most of the studies in Table 3 utilize time-frequency analysis methods such as Fast Fourier Transform (FFT) or wavelet analysis to extract signal characteristics.^7^^,^^69^^,^^110^ FFT is suitable for quickly and efficiently obtaining frequency distribution information of signals, especially for stationary or transient signal analysis. For example, in,^109^ a low-pass Butterworth filter was used in a MATLAB program to obtain real-time time-domain induced voltage from energy-harvesting sensors and FFT was applied to acquire spectral characteristics. Additionally, wavelet time-frequency transformation of acceleration data from corrugated and non-corrugated rails was performed to compare sensitive mutation points in wavelet spectrograms, thereby determining the health status of the tracks.

Different types of DL can be adapted to various functions. In image data processing, CNNs are primarily used for feature extraction in computer vision (CV), demonstrate strong generalization capabilities for classification tasks across different scenarios. Zheng et al. proposed a spatiotemporal enhanced (STE) CNN, which emphasizes spatial responses of foreign objects through feature-level spatiotemporal coherence, facilitating preventive maintenance of high-speed railway catenary systems.^39^ Thus, neural networks can effectively extract features from images for learning and recognition, achieving more precise track detection.^112^

However, when processing collected signals, external sensors are always susceptible to environmental interference factors such as weather and structural changes in facilities. Therefore, utilizing self-sensing signals generated by self-powered devices, such as voltage or power time series, is also feasible. Since track infrastructure directly impacts railway safety, integrating all functions into self-powered devices can save physical space for sensor installation. Moreover, deploying algorithm applications solely through self-powered and self-sensing equipment can avoid the impact of intrusive devices on train operations and the regular maintenance of surrounding track infrastructure.

**Figure 8:**
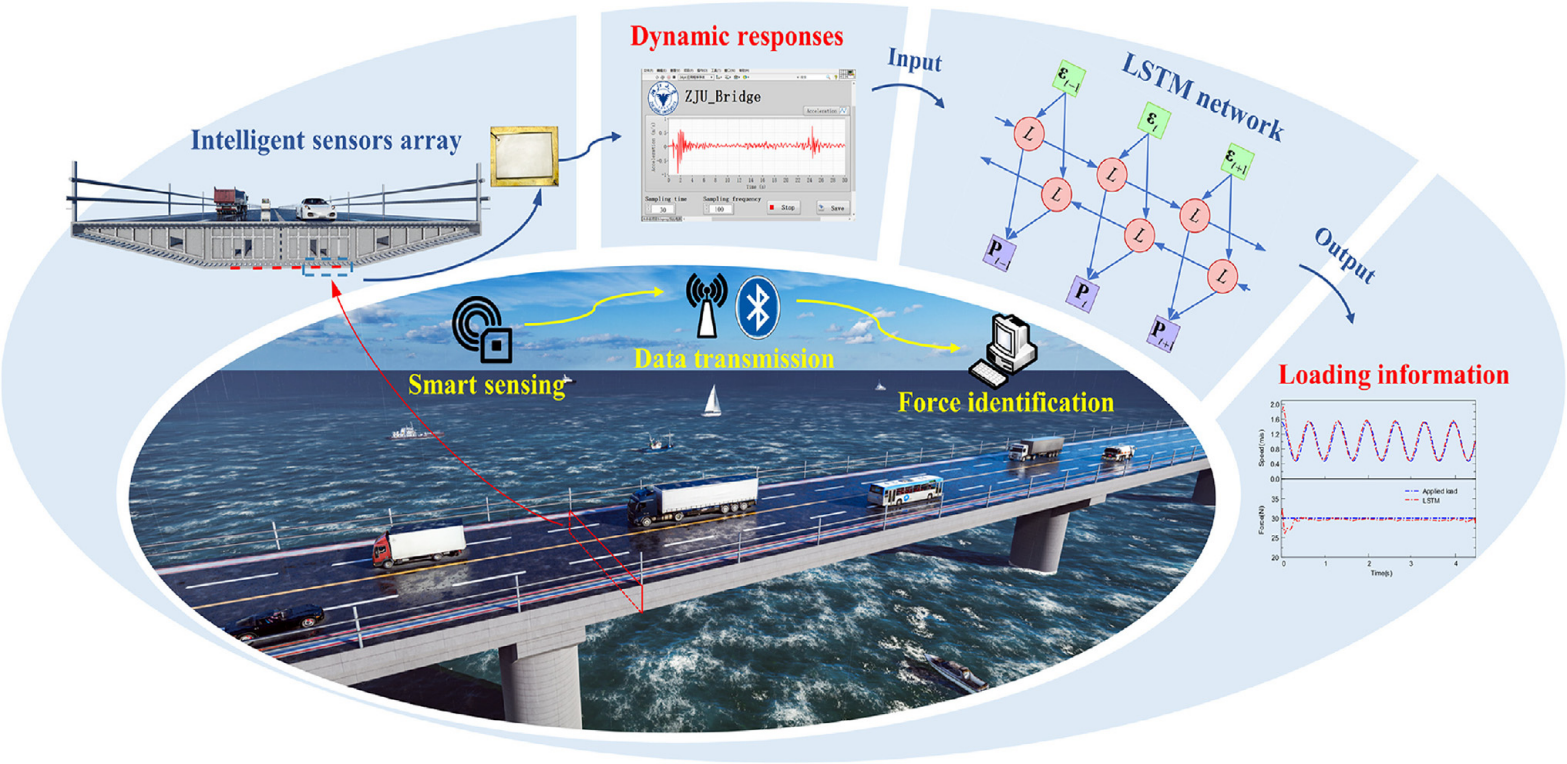
Self-powered devices with different algorithms in railway scenarios LSTM model for detection of bridge structure. (Elsevier Publishing.^119^ Reproduced with permission. All rights reserved.).

In addition to tracking infrastructure, numerous algorithmic studies have been deployed in self-powered systems to detect other railway infrastructure. For example, Wang et al.^111^ utilized EMG and employed stacked denoising autoencoder (SDA) algorithms to reconstruct voltage data affected by noise and obtain robust feature representations. The SDA involves unsupervised pre-training and supervised fine-tuning processes, using trained data to detect transformer faults in track environments. Various environmental monitoring methods are also available for self-powered self-sensing devices installed on bridges.^113^ In Figure 8, Zhang et al.^114^ used three-dimensional finite element analysis and LSTM algorithms to detect bridge structures traversed by railway lines, so did Alavi et al.^115^ Additionally, in specific scenarios such as stations where multiple trains require anti-collision measures, several studies have employed methods such as Balise modeling,^116^ linear fitting,^117^ CNN,^118^ and collision algorithms^84^ to identify vehicle information and perceive train positions, thus achieving collision avoidance around the train bodies.

### State detection of rail vehicles

Despite significant advancements in monitoring systems for rail transit, other systems for the condition of rail vehicles like freight trains, still need to meet the required performance levels.^120^ Due to the complexity of train equipment, train accidents are often attributed to the wear and fatigue of vehicle components.^114^ Real-time continuous monitoring of train conditions can detect component failures early, enhancing rail transit’s efficiency and safety. To avoid disrupting the existing equipment layout, many studies propose utilizing self-powered devices to improve the overall reliability of detection systems. By incorporating algorithms to further utilize sensor data from various components, a more rational analysis of real-time data from different parts of the train can be achieved.

**Figure 9:**
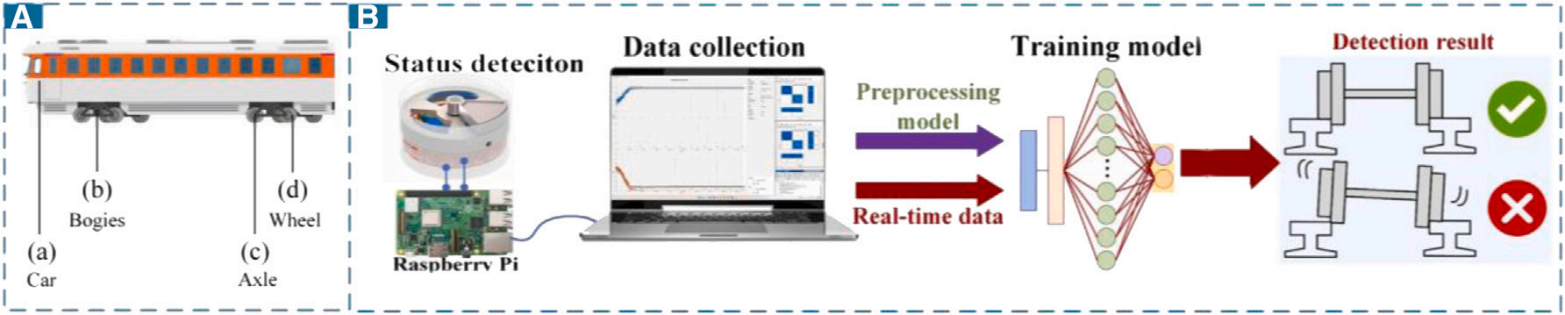
Algorithms for state detection of rail vehicles (A) Different components in rail vehicle. (B) LSTM model for bearings detection in self-powered devices. (Elsevier Publishing.^123^ Reproduced with permission. All rights reserved.).

As previously mentioned, bogies, axles, and wheels generate substantial mechanical energy during operation which can be harvested, shown in Figure 9A. However, these critical and frequently worn components are the focus of health monitoring and stability testing. The train bogie serves as the sole connection between the train body and the tracks and plays a crucial role in ensuring the safe operation of trains. For example, He et al.^89^ analyzed voltage signals generated by an energy management structure (EMI) based on EMG, using strategic algorithms to detect axle cracks. Furthermore, some studies have installed resonant electromagnetic vibration energy harvesters (REVEHs) on bogies. These systems collect and analyze vibration information to provide real-time diagnostics and monitoring of freight trains.^121^ Huang et al.^122^ utilized a one-dimensional CNN (1-D-CNN) to extract features from collected vibration signals, achieving effective fault classification and fault component localization. In addition to CNNs, LSTM algorithms can also analyze and predict data sequences generated during train operation, such as bearing wear patterns or vibration signals. In Figure 9B, Fang et al.^123^ deployed an LSTM network within a self-powered device in the bearings to detect abnormalities and assess the stability of the axles. LSTMs also help predict maintenance times, reducing unexpected downtime and enhancing transportation efficiency and safety. Other studies^124^^,^^125^ have analyzed self-powered signals from axles for monitoring purposes in smart urban transportation scenarios. As the direct contact between train wheels and tracks, they are subject to frequent wear and tear. Therefore, it is equally important to monitor the condition of the wheels and their bearings.^126^ Bernal et al.^127^ proposed a fault detection system based on analog signal processing to identify flat defects in wheels using simulated acceleration signals. This approach reduces memory requirements and power consumption. Other studies have utilized time-frequency analysis of signals from self-powered devices near the wheels for monitoring and predicting wheel conditions.^92^^,^^95^^,^^128^ gathered self-sensing vibration signals and analyzed their correlation with noise data to detect dynamic behaviors of trains, such as anomalies caused by wheel defects.

In addition to monitoring specific components, other data related to rail vehicles should also be focused. For instance, Zanelli et al.^13^ utilized solar power on freight train bodies to intelligently measure pressure values at specific points in the system, including monitoring pressure in the main pipeline and brake cylinders to ensure safety. Monitoring the power generation efficiency of self-powered devices during train operation is also crucial. Some studies have employed modeling and DNN algorithms to predict onboard devices’ voltage and power generation, indirectly analyzing train operating conditions.^129^^,^^130^ Tian et al.^131^ used the LSTM network with good coupling and high prediction accuracy to predict the load of photovoltaic power generation, thereby improving the stability of integrating photovoltaic power into rail transit systems. In intelligent transportation scenarios, research on self-powered and self-sensing devices and algorithms for vehicle condition monitoring is also evolving. These systems are becoming increasingly comprehensive, employing time-frequency analysis and DL algorithms to monitor various data types such as speed, acceleration, and flow.^67^^,^^132^^,^^133^ Due to commonalities in the transportation industry, these improved algorithms also apply to rail vehicles to some extent,^134^ enabling effective real-time monitoring of various vehicle parameters and achieving intelligent applications across different scenarios.

### Behavior analysis of train drivers

In addition to the physical factors of onboard train systems and surrounding infrastructure, human factors such as the driver’s state and behavior also significantly impact train operation safety. Driver fatigue and distraction are particularly influential, accounting for 36% of road traffic accidents.^135^^,^^136^ Li et al.’s assessment of human-machine trust plays a crucial role in developing intelligent transportation automation in high-speed rail automation.^137^ With algorithm advancements, sophisticated human-machine collaborative driving systems typically analyze the driver’s physiological signals to establish dynamic intelligent management modes, thereby enabling intelligent train operation.

**Figure 10:**
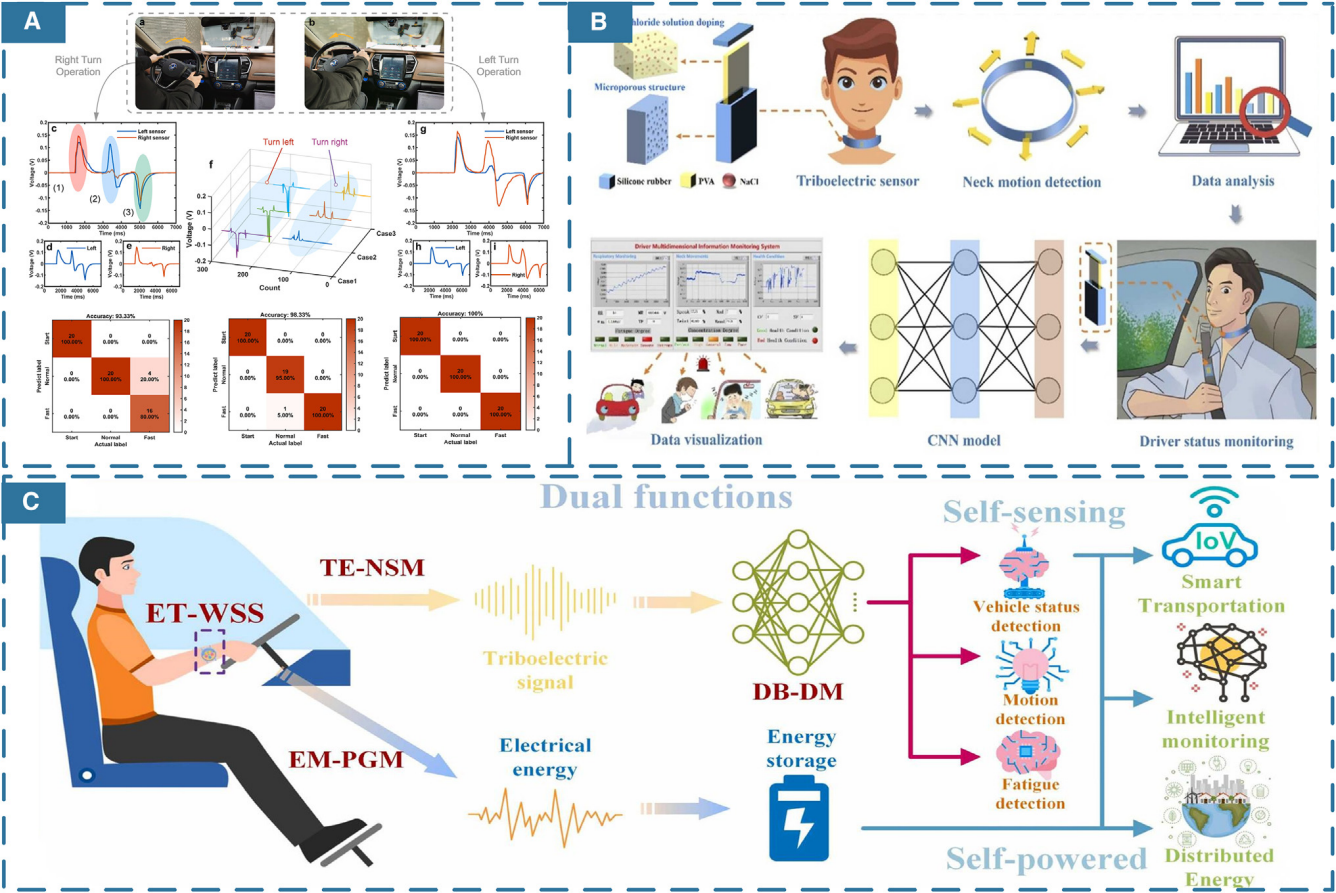
Algorithms applied in Train cab scene (A) MLP for the analysis of voltage sequence signal extraction features. (Elsevier Publishing.^140^ Reproduced with permission. All rights reserved.). (B) CNN is used to monitor driver status. (Elsevier Publishing.^143^ Reproduced with permission. All rights reserved.). (C) Multi-scale RCNN Trained for identifying driver behavior. (Elsevier Publishing.^144^ Reproduced with permission. All rights reserved.).

Like other onboard sensors, self-powered and self-sensing research can be applied to various facilities within the driver’s cabin. However, non-contact sensors such as cameras and infrared cameras often require external power sources and are susceptible to environmental influences. Direct-contact devices with self-powered and self-sensing applications are more suitable instead. A common approach is to mount TENG sensors on the steering wheel to collect energy and signals.^138^ Chen et al.^139^ utilized a multilayer perceptron (MLP) to extract features from voltage sequence signals, indirectly identifying and classifying driver behaviors. Additionally, the brake pedal which is operated for extended periods, also holds potential for energy harvesting and information collection. Some studies^140^^,^^141^ have mounted TENG sensors on moving brake pedals and used FFT algorithms to analyze driver behavior, shown in Figure 10A. Zhang et al.^142^ proposed a driver training assistance system (DTAS) which integrates three triboelectric driving operation sensors, including gear shift sensor, steering angle sensor, and pedal sensors. He also employed a CNN network, which is particularly effective at extracting subtle features from time-series data, to learn signals generated by the brake pedal, detecting and analyzing drivers' driving habits.

However, indirect signal collection through other facilities cannot accurately monitor the driver’s physiological signals. The development trends of advanced smart transportation emphasize the importance of incorporating human factors engineering in vehicle ergonomics. Human factors engineering provides interdisciplinary perspectives for developing driver monitoring systems, including wearable devices and ML algorithms, to enhance safety and comfort.^145^ Luo et al.^143^ designed a green, stretchable triboelectric sensor mounted on a wearable neck ring shown in Figure 10B. The neck ring was placed in safety to collect signals generated by neck muscle movements, including turning the head, coughing, speaking. With K-nearest neighbor (KNN), SVM, and CNN algorithms, the smart neck ring also classified movements to detect driver fatigue and other dangerous driving behaviors. In Figure 10C, Chen et al. and Yang et al.^144^^,^^146^ both designed wearable wristband devices as energy harvesters and signal collectors. Chen et al.’s approach involved training self-sensing signals with a multi-scale RCNN algorithm, resulting in accurate recognition of driver behaviors and enhancing the reliability of human-machine interaction in driving systems. New research indicates that there are other effective methods for monitoring physiological signals that minimize discomfort to the driver. Zhang et al.^147^ employed a ring device using an LSTM algorithm to recognize driver gestures and actions. It demonstrates the practical application of human factors engineering in self-powered and self-sensing wearable devices for transportation scenarios.

### Optimization and control of rail transit devices

In addition to passively recording sensor data from various rail transit scenarios, self-powered devices should actively participate in optimizing and controlling trains and other infrastructure.^148^ utilized the gray wolf optimization and particle swarm optimization (GWO-PSO) algorithms to develop finite element analysis models for optimizing self-powered devices mounted on freight train suspension dampers. A vehicle-track coupling model was established to predict more accurate vibration responses and enhance the vibration energy harvesting efficiency of the train suspension during operation. Similarly, Fu et al. applied FFT analysis to sensor data collected from vibration energy harvesters on train bogies.^149^ They revealed changes in the main vibration frequency in both the time and frequency domains to determine the optimal vibration frequency of the devices. Lopes et al.^70^ employed an interactive adaptive weighted genetic algorithm (I-AWGA) to ascertain the optimal geometric shape of the devices, capitalizing on the algorithm’s adaptability, parallelism, and global search capability. These studies aim to maximize energy harvesting through algorithmic design.

**Figure 11:**
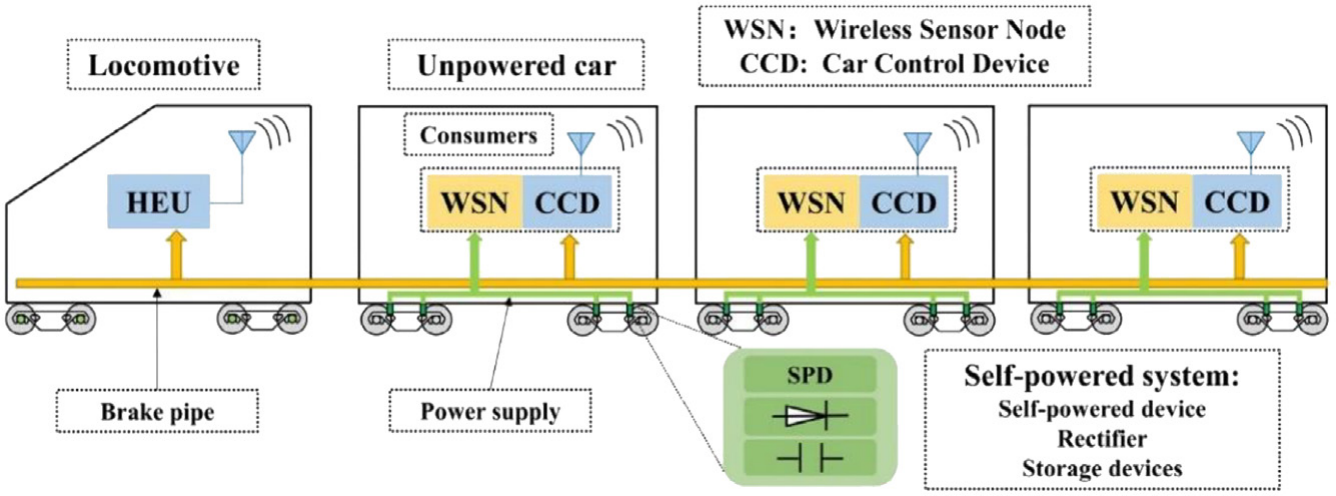
Optimizing control algorithms algorithm in railway scenes A vehicle-track coupled model for the excitation analysis. (Elsevier Publishing.^150^ Reproduced with permission. All rights reserved.).

In optimizing train control systems during operation, Zuo et al.^150^ developed an electro-mechanical coupling model based on self-powered PENG to predict performance under varying load conditions and train speeds shown in Figure 11. This model was subsequently utilized to control train braking. Guo et al.^151^ proposed a promising speed-sensing and braking monitoring solution. It employs control algorithms to manage the electronic control unit (ECU) for speed control. Advanced intelligent control systems are crucial in ensuring vehicle safety at critical moments.

In addition to device and control optimization, algorithms also play a role in the structural optimization of trains. For instance, Lian et al.^152^ utilized LSTM models to characterize the nonlinear functions of data relationships within the system. This enabled adaptive parameter control of the high-speed railway subgrade vibratory rolling compaction process for enhanced structural stability. Sun et al.^153^ developed a mathematical model for the suspension system of medium- and low-speed maglev trains by employing an improved *a priori* algorithm to analyze stored historical data and determine adaptive fuzzy rules for the maglev train suspension system.

### Energy management of rail transit devices

The energy requirements of general sensors can be adequately met due to the development of self-powered devices. However, the excess energy collected necessitates self-powered devices to possess energy storage and management capabilities. Some studies have utilized control strategies^154^ and frequency domain analysis^155^ to manage the power flow of self-powered devices effectively, achieving energy balance. Morais et al.^156^ optimized the efficiency of charging controllers in railway transportation systems using fuzzy logic control and genetic algorithm metaheuristics to adjust fuzzy rule weights automatically. Intelligent energy management is essential in long-term rail transit operations, especially when energy harvesters temporarily cease functioning.

**Figure 12:**
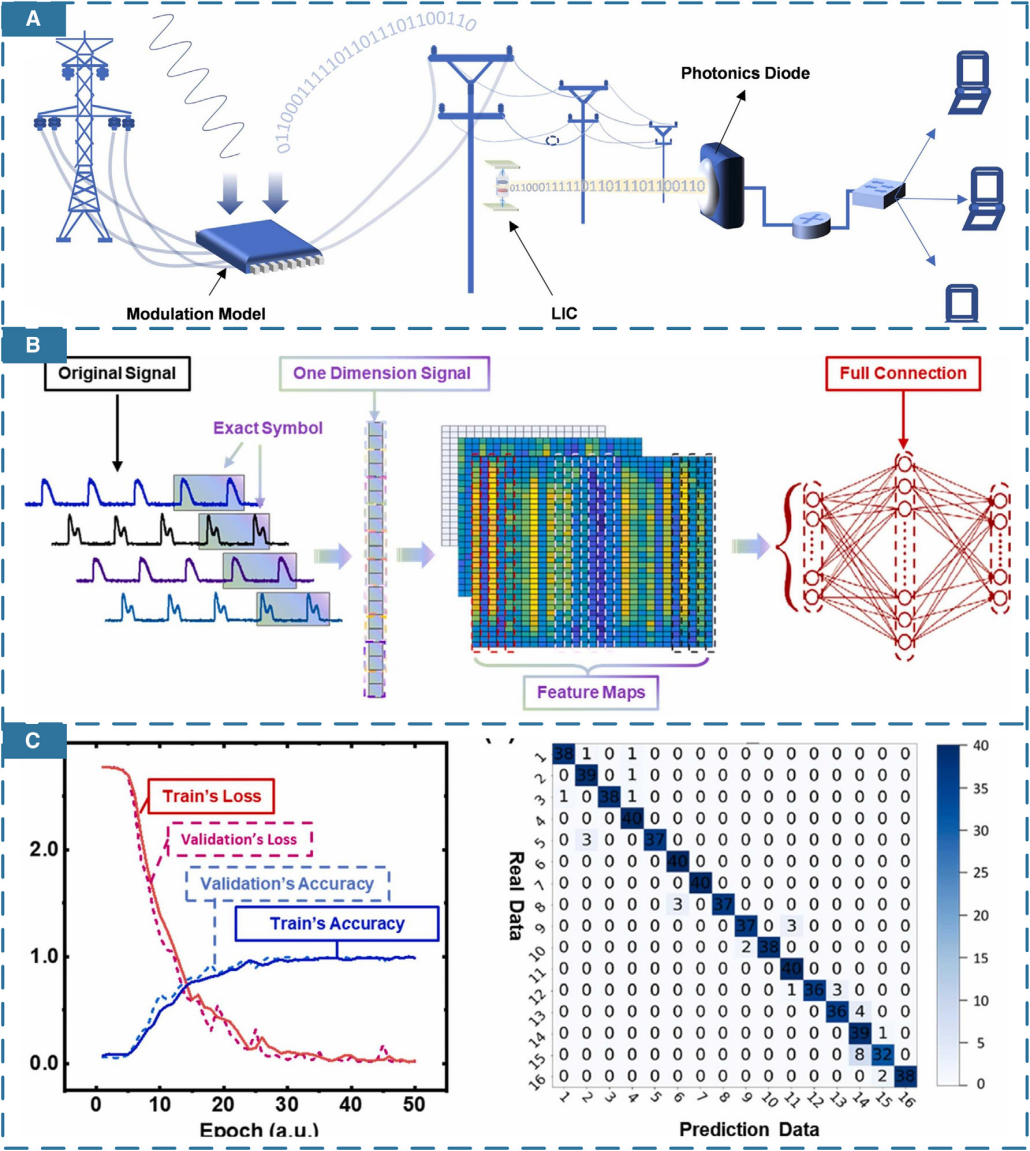
smart self-powered high-voltage monitoring (Elsevier Publishing.^158^ Reproduced with permission. All rights reserved.). (A) The structure of a self-powered power-line monitoring system. (B) CNN network applied in the signals analysis. (C) Training and validation results of identifying harmonic pollution.

Furthermore, for increasingly intelligent power grid systems, the transmission of surplus energy from rail transit to smart grids for unified algorithmic management can comprehensively monitor grid status and operating conditions.^157^ Shown in Figures 12A–12C, Liao et al. applied CNN algorithm to accurately demodulate the high-frequency carrier signals transferred in the high-voltage line.^158^ In rail environments with distributed self-powered systems, using microgrids and distributed grids can enhance the power system’s resilience, effectively addressing potential issues such as sudden power outages.^159^

With the advancement of self-powered sensors in rail transit, it is highly logical to leverage ML algorithms to utilize self-sensing data. Whether monitoring railway environments or vehicle conditions, these algorithms can enhance detection accuracy and add new functionalities. Such improvements support the intelligent development of rail transit under the industry 4.0 framework. Moreover, optimization algorithms for devices and energy enhance the adaptability and robustness of self-powered devices, ensuring sustainable operation and providing a foundation for green railways. Similar to autonomous driving technology on roads, human factors engineering research using self-powered self-sensing technology for train drivers also contributes to automation in rail transit.

There are still many other algorithms active in rail transit. The transfer learning of DL has especially facilitated the application of advanced algorithms in cross-disciplinary fields such as self-powered and self-sensing technology. As discussed in this section, self-powered devices can harness abundant renewable energy in rail transit scenarios, and utilize algorithms integrated with IoT technology to document the energy harvesting process. Cross-disciplinary applications can effectively leverage all available information in these scenarios, providing ample data and foundational algorithm logic for the system’s intelligence. The utilization of algorithms meets the data-driven complex detection and decision-making requirements of Industry 4.0^160^ and showcases the integration of other emerging technologies in rail transit.

## Opportunities for emerging technologies

In addition to addressing the smart development needs of Industry 4.0 in AIoT and AI algorithms, Industry 5.0 introduces a human-centric concept that emphasizes technology development, prioritizing human well-being, social progress, and environmental sustainability. This section focuses on emerging technologies in rail transit, including advancements in human-machine interaction, bidirectional analysis of digital-physical spaces, and improvements toward high-speed, high-capacity trains.

### Large language models (LLMs) for human-machine interaction

As mentioned before, AI-based methods can automatically analyze data and provide accurate predictions by learning from historical data. Particularly, recent revolutionary advancements in large language models (LLMs) have good performance in dealing with natural language processing (NLP).^161^ Wang et al.^162^ proposed integrating LLMs with sensor networks and monitoring systems to analyze operational historical data and equipment status, thus generating corresponding alerts. LLMs can also be utilized to develop intelligent operator assistance systems in order to offer real-time, precise recommendations and decision support, thus facilitating human-machine collaboration advocated by Industry 5.0. Due to their characteristics, LLMs can create domain-specific knowledge bases and use instructions or prompts for fine-tuning. These operations both allow customized task handling in different scenarios.

As the LLM algorithm becomes the frontier research, there is currently a scarcity of literature related to self-powered sensing or IoT studies. Nevertheless, LLM holds significant potentials in rail transit, particularly in predictive maintenance and health management (PHM) systems which includes real-time monitoring and assessment of railway equipment as well as forecasting potential failures.^163^ In the previously discussed self-powered sensing and IoT framework, conditions such as abundant energy supply and intelligent sensor management have facilitated the widespread implementation of PHM in rail transit through other ML or DL algorithms. Consequently, it is believed that LLM can be well integrated into self-powered sensing systems in the future to achieve advanced PHM in rail transit.

### Digital twin (DT) for the virtual

Digital twin (DT) technology gathers extensive data from physical spaces and conducts thorough analyses of entities. It aims to generate complete descriptions and display them in the information space, in order to enable precise remote management.^164^^,^^165^ In rail transit, DT technology can develop dynamic, personalized digital simulations of physical trains, allowing for real-time applications such as predictive maintenance, adaptive optimization, and fault detection.^166^^,^^167^^,^^168^ Liu et al.^169^ established a DT framework to populate historical data of train operations for fault diagnosis. He also utilized virtual reality mapping within the DT framework to optimize the dynamics model of train bearings. Like most technologies developed under the AIoT framework, DT also integrates ML or DL algorithms to make data-driven intelligent decisions, such as pattern recognition and predictive analysis in virtual environments. Wu et al.^170^ proposed a DT-based bogie fault diagnosis framework that integrates seven dimensions of data (from physical equipment to supplier networks and utilizes a multi-layer CNN data-driven model for fault diagnosis to address the challenge of real-time monitoring of bogies. Bosso et al.^171^ employed SVM algorithms for multibody simulations within the DT framework. By combining both techniques, modeling capabilities for train dynamics analysis can be enhanced.

Although the literature about DT technology with self-powered sensing systems is limited, numerous studies have focused on enhancing rail safety, maintenance, and efficiency through the DT framework. Wang et al.^172^ analyzed the necessity for DT technology in traction power supply systems and provided a comprehensive discussion on its application modes for energy conservation. The article highlights that DT can facilitate the self-evolution and updating of rail or track models. Therefore, it enables the collection of status data from various dimensions, thereby achieving parallel control over energy consumption and operations with high reliability and efficiency in rail transit.

### High-temperature superconducting (HTS) and magnetic levitation (maglev) for high-speed railways

**Figure 13:**
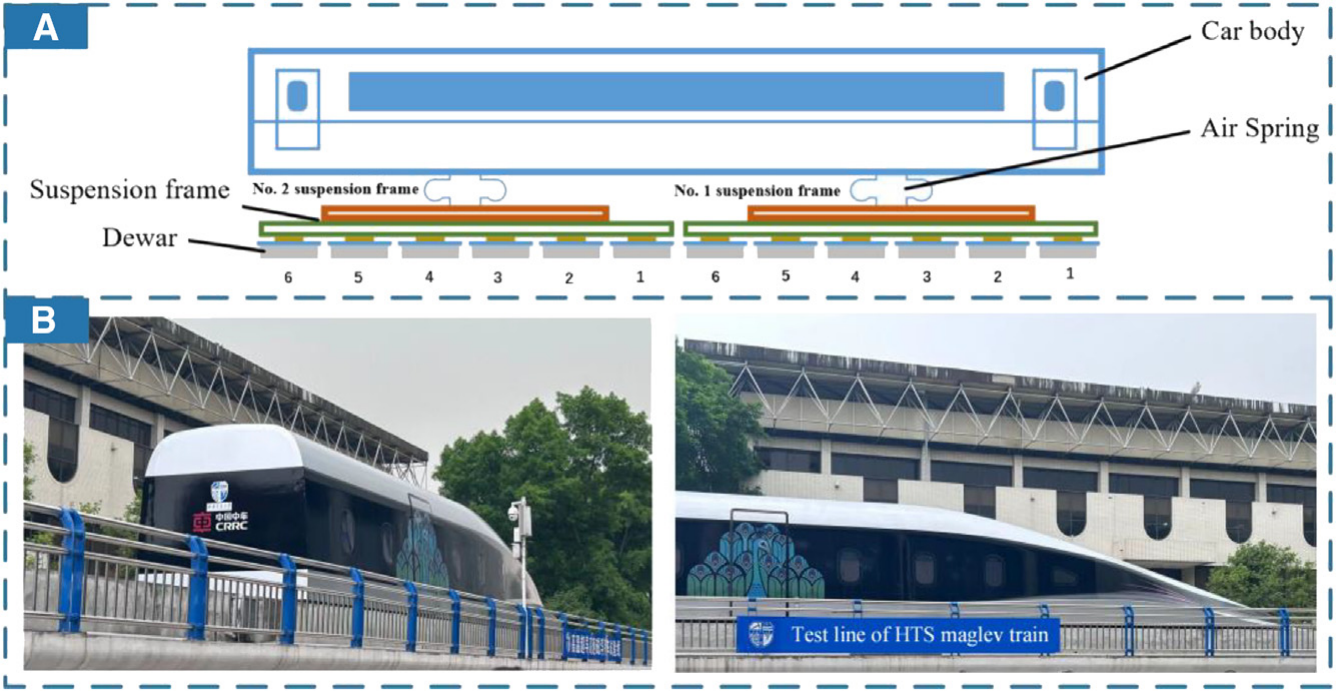
Advanced applications of HTS maglev train (A) Vertical structure diagram of HTS Maglev vehicle. (Elsevier Publishing.^176^ Reproduced with permission. All rights reserved.). (B) A test line of HTS maglev train in Southwest Jiaotong University.

As materials science develops, the increasingly mature high-temperature superconducting (HTS) technology has begun to be applied in rail transit scenarios, due to its excellent energy transmission characteristics.^173^ Allais et al.^174^ proposed the SuperRail project, which involves the development, manufacturing, installation, and long-term operation of HTS direct current cable systems for railway applications. It aims to enhance the power supply in the train station. Magnetic levitation (maglev) technology based on HTS materials can enable trains to maintain stable levitation without any control system or operational speed.^175^ HTS maglev vehicle models do not require onboard traction power devices or wheel traction adhesion, making the vehicles relatively lightweight and demonstrating significant potential in high-speed rail transit. In Figure 13A, Fu et al.^176^ discussed how the performance of vehicle-track coupled vibrations affects vehicle operational stability and provided reasonable recommendations to improve the safety of such high-speed trains. Furthermore, control and optimization algorithms are also applicable to trains supported by this technology. Li et al.^177^ employed a fuzzy-PID algorithm to control the secondary suspension of HTS maglev vehicles, enhancing ride comfort and offering better insights for ergonomic development in high-speed railway applications. Figure 13B shows the practical achievements of HTS maglev technology so far.

## Conclusion and challenges

As the global railway network expands and develops, self-powered and self-sensing devices have become increasingly effective in various rail transit scenarios. This review offers a comprehensive review of the development characteristics of autonomous energy harvesting devices in the railway industry. It summarizes the current features and implementation methods of self-powered devices from multiple perspectives, including power generation methods, material selection, and application scenarios. Additionally, it analyzes these devices within the existing IoT framework, discussing the self-sensing capabilities enabled by their integration with IoT functionalities such as sensing, communication, and computation. Through extensive research discussions, it is evident that by using the network layer in the IoT architecture as a bridge, mechanical devices at the physical layer can be combined with various advanced algorithms at the application layer. All the examples showcased the potential and possibilities of self-powered and self-sensing devices in rail transit.

The results indicate that continuous iteration and improvement of cutting-edge technologies (particularly DL algorithms) have provided comprehensive solutions to issues in rail transit. These methods enhance the utilization of sensor signals generated by the devices and offer mature AIoT solutions that combine IoT with rail transit. Therefore, the integration of self-powered and self-sensing devices with advanced technologies significantly addresses existing challenges in rail transit, especially for autonomous monitoring and the collection, processing, and transmission of complex signals in the plateau, remote mountainous and high-cold regions. However, the development of self-powered sensing technologies still faces several challenges, particularly in the integration of diverse technologies. Considering both spatial and economic costs, the coordination between self-powered devices and embedded systems (e.g., IoT communication modules or AI chips) has to be integrated into the overall design. As the researches integrate multiple technologies, the lack of coordination across different domains tends to be serious.

**Figure 14:**
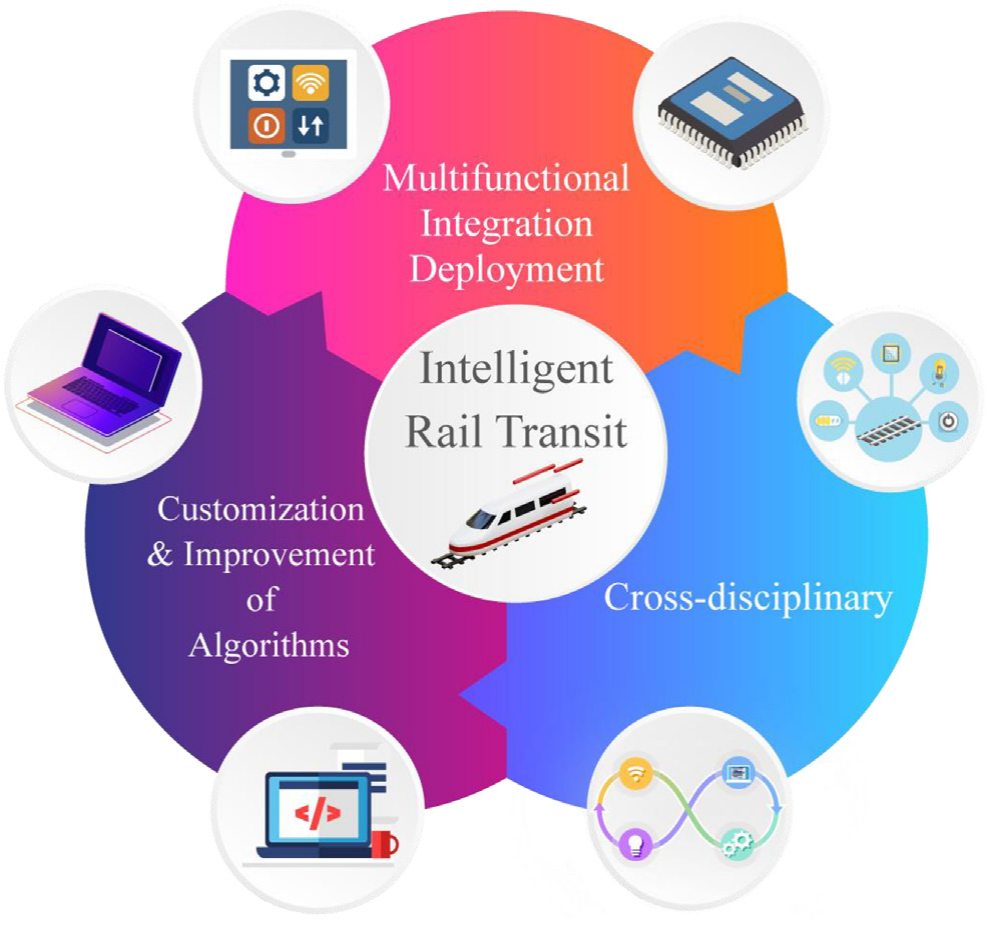
Prospects of sustainable and smart rail transit

Consequently, we have identified several key points and explored some emerging research directions, ultimately providing supplementary solutions and broader perspectives for the intelligent development of rail transit, shown in Figure 14.

### Multifunctional deployment integration

Current self-powered technologies have significantly improved power generation efficiency and expanded power generation methods. Those devices that solely function as power sources are insufficient for adapting to changing environments and lack practical value. Although this review mainly focuses on scenarios involving self-powered applications, the number of related studies decreases as functionalities increase. Therefore, there is a growing emphasis on integrating multiple functions into a single self-powered device, including self-sensing, device optimization control, data communication, and algorithm deployment. Notably, advanced self-powered devices can integrate IoT and AI functionalities, such as those under the TinyML framework, via MEMS technology without compromising power efficiency.^178^ In the environment of rail transit, the development direction of self-powered technology will continue to integrate and deploy multiple functions,^179^ so as to assimilate and adapt to more advanced technologies.

### Customization and improvement of technologies and algorithms

As the railway environment becomes increasingly complex and variable, the energy and data sources collected by self-powered and self-sensing devices also become more diverse and heterogeneous. Single method in research have shown limitations, such as solar or wind energy collectors failing to function in adverse weather conditions. Specific hybrid energy harvesting methods can be implemented to address other effective energy sources in the environment.^180^ It ensures that at least one usable resource is available for normal monitoring and maintenance of rail transit. Specific algorithm improvements are needed for issues such as the varying quality and incompleteness of collected information. Improvements include introducing noise and bias, lightweight models, and multi-modal information fusion.^181^ Lightweight models can increase the feasibility of deployment on embedded edge devices. At the same time, multi-modal information fusion methods can combine rich signals collected from the environment, enhancing the robustness of rail monitoring and vehicle status monitoring tasks.

### Cross-disciplinary

Some studies have already integrated rail self-powered and self-sensing technology with DL algorithms. Other technologies like wearable design^182^ and materials science^183^ indicate that interdisciplinary applications remain a prevailing trend in technology development. Numerous studies have demonstrated that specific problems within a single field can be too complex and demanding to solve effectively. However, current research efforts can receive direct assistance by combining tools from other fields. Both algorithms aiding in data processing and more theoretical approaches to material structure design can facilitate better optimization of the installed devices in the rail environment. Furthermore, advanced analytical methods from other fields can enhance research efficiency through approaches similar to transfer learning.

## Acknowledgments

This work was supported by the National Natural Foundation of China under Grants No. 51975490 and by the Science and Technology Projects of Sichuan under Grants Nos. 23QYCX0280 and 2022NSFSC0461; and by the Science and Technology Projects of Yibin under Grant No.2021ZYCG017, 2023SJXQYBKJJH005, No. SWJTU2021020001 and SWJTU2021020002; and by the Science and Technology Projects of Chengdu under Grant No. 2021YF0800138GX.

## Author contributions

H.T.: Methodology, Software, Writing - Original Draft. L.K.: Investigation, Resource, Writing – Review and Editing. Z.F.: Investigation, Resource, Formal analysis. Z.Z.: Supervision, Project administration, Funding acquisition. J.Z.: Formal analysis, Writing – Review and Editing. H.C.: Investigation, Resources. J.S.: Resources. X.Z.: Resources.

## Declaration of interests

The authors declare that they have no known competing financial interests or personal relationships that could have influenced the work reported in this review.
